# Supplementary material for: MS CD49d+CD154+ Lymphocytes Reprogram Oligodendrocytes into Immune Reactive Cells Affecting CNS Regeneration
Source: Cells. 2019 Nov 25;8(12):1508. doi: 10.3390/cells8121508 (PMC6953114; doi:10.3390/cells8121508)
Supplement: Supplementary file 1 [file cells-08-01508-s001.pdf]

Supplementary Table 1. A list of individual putative targets of the hsa-miR-665, hsa-miR-21-3p and hsa-miR-212-3p. The mirSVR scores were used to classify the most important target of miRNA which had the strongest affinity and the best compatibility to a particular mRNA.

Gene symbol	Gene description and protein function	mirSVR score
	hsa-miR-665	
BTAF1<br>NM_003972	BTAF1 RNA polymerase II, B-TFIID transcription factor-associated	-2.30
LRCH3<br>NM_032773	leucine-rich repeats and calponin homology (CH) domain containing 3	-2.01
NIPBL<br>NM_133433	Nipped-B homolog, Nipbl protein is required for the association of cohesin with DNA	-2.01
CDH18<br>NM_004934	cadherin 18 type 2; CDH18 play roles in cell adhesion, forming adherents junctions to bind cells together within tissues	-1.98
COL3A1<br>NM_000090	Collagen type III, alpha 1	-1.86
EXT1<br>NM_000127	exostoses (multiple) 1; protein - endoplasmic reticulum-resident type II transmembrane glycosyltransferase involved in the chain elongation step of heparan sulfate biosynthesis	-1.75
	hsa-miR-21-3p	
CLCA3P<br>NR_024604	chloride channel accessory 3 pseudogene; transcribed pseudogene belonging to the calcium sensitive chloride conductance protein family	-2.63
SATB1<br>NM_002971	SATB homeobox 1; global chromatin organizer and transcription factor	-2.48
MALT1<br>NM_006785	mucosa associated lymphoid tissue lymphoma translocation gene 1	-2.34
SLC35F5<br>NM_025181	solute carrier family 35, member F5	-2.33
C17orf104<br>NM_001145080	chromosome 17 open reading frame 104; stabilizes early meiotic RNA messengers; inhibits the degradation of meiotic transcripts and binds to RNA helicase along with several meiotic mRNAs	-2.18
ZNF367<br>NM_153695	zinc finger protein 367; inhibits cellular proliferation, invasion, migration, and adhesion to extracellular proteins	-2.17
PRTFDC1<br>NM_020200	phosphoribosyl transferase domain containing 1; catalyzes the conversion of hypoxanthine and guanine to their respective nucleoside monophosphates.	-2.07
VPS8<br>NM_015303	vacuolar protein sorting 8; plays a role in vesicle-mediated protein trafficking of the endocytic membrane transport pathway	-2.05
	hsa-miR-212-3p	
GTF2H1<br>NM_005316	general transcription factor IIH, polypeptide 1, 62kDa	-2.85
SIRT1<br>NM_012238	sirtuin (silent mating type information regulation 2 homolog) 1; regulates epigenetic gene silencing and suppresses recombination of rDNA	-2.71
MIER1<br>NM_020948	mesoderm induction early response 1 homolog (Xenopus laevis)	-2.59
MIS12<br>NM_024039	MIND kinetochore complex component, homolog (S. pombe); MIS12 complex is required for kinetochore formation during mitosis and normal chromosome alignment and segregation	-2.45
PHF20L1<br>NM_016018	PHD finger protein 20-like 1; supports chromatin loading of DNMT1 and also protects it from proteasomal degradation	-2.38
CCDC150<br>NM_001080539	coiled-coil domain containing 150	-2.38
AZIN1<br>NM_015878	antizyme inhibitor 1; plays a role in cell growth and proliferation by maintaining polyamine homeostasis within the cell	-2.22
LOC401074<br>BC039495	hypothetical LOC401074	-2.18
